# Supplementary material for: scooby: modeling multimodal genomic profiles from DNA sequence at single-cell resolution
Source: Nat Methods. 2025 Oct 22;22(11):2275–85. doi: 10.1038/s41592-025-02854-5 (PMC12615262; doi:10.1038/s41592-025-02854-5)
Supplement: Supplementary file 1 — Supplementary Fig. 1. [file 41592_2025_2854_MOESM1_ESM.pdf]

nature methods

Supplementary information https://doi.org/10.1038/s41592-025-02854-5

scooby: modeling multimodal genomic profiles from DNA sequence at single-cell resolution

In the format provided by the authors and unedited

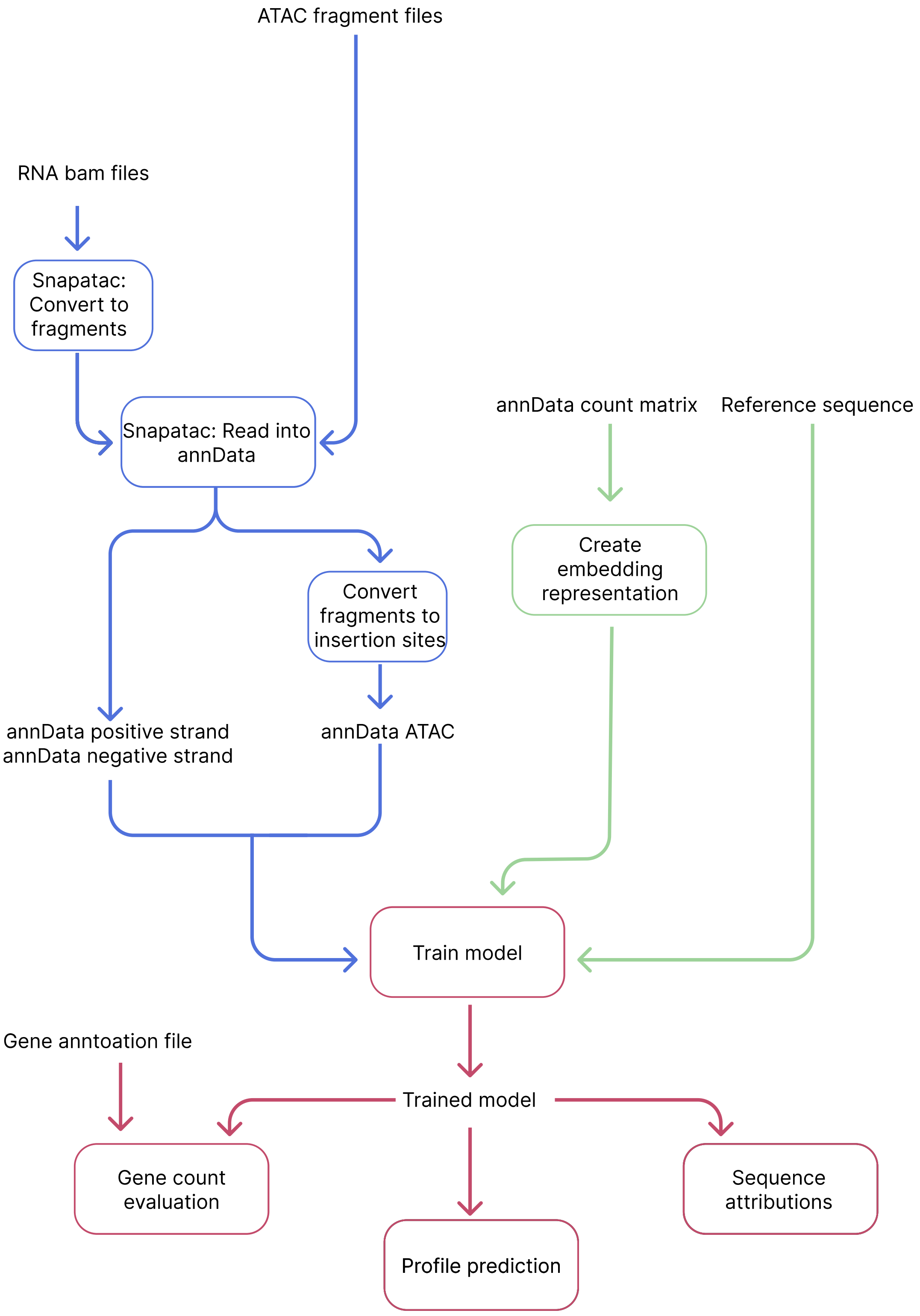

Supplementary Fig. 1: scooby framework flowchart. Diagram illustrates the workflow for generating cell embeddings and preparing RNA-seq and ATAC-seq data for training, including filtering, embedding generation, and coverage extraction.
