# Supplementary material for: scooby: modeling multimodal genomic profiles from DNA sequence at single-cell resolution
Source: Nat Methods. 2025 Oct 22;22(11):2275–85. doi: 10.1038/s41592-025-02854-5 (PMC12615262; doi:10.1038/s41592-025-02854-5)
Supplement: Supplementary file 2 — Reporting Summary [file 41592_2025_2854_MOESM2_ESM.pdf]

nature portfolio

Corresponding author(s): Julien Gagneur

Last updated by author(s): May 21, 2025

Reporting Summary

Nature Portfolio wishes to improve the reproducibility of the work that we publish. This form provides structure for consistency and transparency in reporting. For further information on Nature Portfolio policies, see our Editorial Policies and the Editorial Policy Checklist.

Statistics

For all statistical analyses, confirm that the following items are present in the figure legend, table legend, main text, or Methods section.

n/a	Confirmed	
☐	☒	The exact sample size (*n*) for each experimental group/condition, given as a discrete number and unit of measurement
☒	☐	A statement on whether measurements were taken from distinct samples or whether the same sample was measured repeatedly
☐	☒	The statistical test(s) used AND whether they are one- or two-sided<br>*Only common tests should be described solely by name; describe more complex techniques in the Methods section.*
☒	☐	A description of all covariates tested
☐	☒	A description of any assumptions or corrections, such as tests of normality and adjustment for multiple comparisons
☐	☒	A full description of the statistical parameters including central tendency (e.g. means) or other basic estimates (e.g. regression coefficient) AND variation (e.g. standard deviation) or associated estimates of uncertainty (e.g. confidence intervals)
☐	☒	For null hypothesis testing, the test statistic (e.g. *F*, *t*, *r*) with confidence intervals, effect sizes, degrees of freedom and *P* value noted<br>*Give P values as exact values whenever suitable.*
☒	☐	For Bayesian analysis, information on the choice of priors and Markov chain Monte Carlo settings
☒	☐	For hierarchical and complex designs, identification of the appropriate level for tests and full reporting of outcomes
☐	☒	Estimates of effect sizes (e.g. Cohen's *d*, Pearson's *r*), indicating how they were calculated

Our web collection on statistics for biologists contains articles on many of the points above.

Software and code

Policy information about availability of computer code

Data collection	No software was used to collect data.
Data analysis	We used the following python packages with Python (v3.9.19): scanpy (v1.10), CellRanger ATAC (v2.1.0), Cell Ranger (v8.0.1), Cell Ranger (v6.1.1), pyRanges (v0.0.129), scvi (v1.1.2, https://github.com/lauradmartens/scvi-tools/tree/poissonmultivi), SnapATAC2 (v1.0.1, https://github.com/lauradmartens/SnapATAC2), rustup (v1.28.1), rustc (v1.85.0), scarches (v0.6.1), Borzoi (v0.0.2, https://github.com/johahi/borzoi-pytorch), peft (v0.10.1, https://github.com/lauradmartens/peft), trackplot (v0.4.0), PyTorch (v2.1.0), tangermeme (v0.2.3), pychromvar (v0.0.4), seq2cells (https://github.com/GSK-AI/seq2cells), Unipressed (v1.3.0), Meme suite, scipy(v1.13.1), gseapy (v1.1.3), seq2cells (https://github.com/GSK-AI/seq2cells), jupyterlab (v4.2.0), tomtom (v5.5.2).<br>The scooby model including training scripts and data loaders are available at https://github.com/gagneurlab/scooby. Jupyter notebooks and scripts to reproduce our analysis and figures are available at https://github.com/gagneurlab/scooby_reproducibility. The adapted version of SnapATAC2 is available at https://github.com/lauradmartens/SnapATAC2. The scooby model including training scripts and data loaders are available at https://github.com/gagneurlab/scooby. Jupyter notebooks and scripts to reproduce our analysis and figures are available at https://github.com/gagneurlab/scooby_reproducibility. The adapted version of SnapATAC2 is available at https://github.com/lauradmartens/SnapATAC2. The code along with data to reproduce the findings have additionally been archived and are available on Zenodo at https://doi.org/10.5281/zenodo.15517764 and https://doi.org/10.5281/zenodo.15517072.

For manuscripts utilizing custom algorithms or software that are central to the research but not yet described in published literature, software must be made available to editors and reviewers. We strongly encourage code deposition in a community repository (e.g. GitHub). See the Nature Portfolio guidelines for submitting code & software for further information.

Data

Policy information about availability of data

All manuscripts must include a data availability statement. This statement should provide the following information, where applicable:

- Accession codes, unique identifiers, or web links for publicly available datasets
- A description of any restrictions on data availability
- For clinical datasets or third party data, please ensure that the statement adheres to our policy

The scRNA-seq, scATAC-seq, and pre-processed count matrices for the multiome hematopoiesis dataset are available from the NeurIPS 2021 challenge, SRA (accession SRP356158), AWS (s3://openproblems-bio/public/post_competition/multiome/), and GEO (accession GSE194122). The epicardioids dataset raw data (scATAC-seq, scRNA-seq) is available from SRA (accessions SRP359250, SRP359249). The OneK1K dataset raw data (scRNA-seq) is available from SRA (accession SRP359840). Pre-processed OneK1K data is available from CZ CELLxGENE: https://cellxgene.cziscience.com/collections/dde06e0f-ab3b-46be-96a2-a8082383c4a1. We used the Cell Ranger references refdata-cellranger-arc-GRCh38-2020-A-2.0.0 and refdata-gex-GRCh38-2020-A. We used the GENCODE release v32 GTF file and the GO Biological Process 2021 gene set. TF position weight matrices were obtained from HOCOMOCO v12 (https://hocomoco12.autosome.org/downloads_v12). GTEx eQTL and OneK1K summary statistics and fine-mapping results are available at https://www.ebi.ac.uk/eqtl/. The scATAC-seq PBMC dataset was downloaded from https://app.azimuth.hubmapconsortium.org/app/human-pbmc-atac. The GWAS Catalog (v1.0) was downloaded from https://www.ebi.ac.uk/gwas/docs/file-downloads. We matched SNP IDs using dbsnp (rs_id_dbSNP151_GRCh38p7, https://www.ncbi.nlm.nih.gov/snp/).

Research involving human participants, their data, or biological material

Policy information about studies with human participants or human data. See also policy information about sex, gender (identity/presentation), and sexual orientation and race, ethnicity and racism.

Reporting on sex and gender	n/a
Reporting on race, ethnicity, or other socially relevant groupings	n/a
Population characteristics	n/a
Recruitment	n/a
Ethics oversight	n/a

Note that full information on the approval of the study protocol must also be provided in the manuscript.

Field-specific reporting

Please select the one below that is the best fit for your research. If you are not sure, read the appropriate sections before making your selection.

☒ Life sciences ☐ Behavioural & social sciences ☐ Ecological, evolutionary & environmental sciences

For a reference copy of the document with all sections, see nature.com/documents/nr-reporting-summary-flat.pdf

Life sciences study design

All studies must disclose on these points even when the disclosure is negative.

Sample size	The study utilized three primary single-cell datasets (NeurIPS Hematopoiesis dataset, OneK1K Cohort, Heart Organoid dataset), supplemented by GTEx bulk eQTLs, to comprehensively evaluate scooby's diverse capabilities. Collectively, these datasets were sufficient as they enabled assessment across different biological contexts, scales, analytical tasks (profile modeling, TF activity, eQTL prediction), and allowed benchmarking against existing methods, supporting the paper's main claims.
Data exclusions	Peaks and genes from the datasets were excluded when they had counts in less than 1% of the cells. We identified and removed doublet cell populations in the Neurips dataset using Scrublet with default parameters. Doublet calls were based on a threshold that primarily captured cells clustering in discrete locations on the Uniform Manifold Approximation and Projection (UMAP) embedding. We removed the cell types 'Platelets' and 'Erythrocytes' to retain only immune cell types for the OneK1K dataset. For the epicardioids dataset we retained cells for which we had a scRNA-seq and scATAC-seq match.
Replication	We ensured robust evaluation by following the same sequence-level train and test splits as our underlying foundation model Borzoi. Moreover, genes and scATAC-seq peaks overlapping with validation or test regions were excluded from the input data used to generate the single-cell embeddings to avoid data leakage. No experimental findings were disclosed, hence no replication was performed.
Randomization	Random allocation is not relevant as this is a computational modeling study using pre-existing datasets. Unbiased evaluation is ensured through fixed train/validation/test splits of genomic data, preventing data leakage, even from the underlying Borzoi model. Scooby explicitly

	accounts for key covariates like cell type by conditioning predictions on cell-specific embeddings. Performance is then assessed with objective metrics, often stratified by these known biological factors, ensuring robust and fair evaluation.
Blinding	Blinding of investigators to group allocation during data collection was not relevant to this study. The research involves the development, training, and evaluation of a computational model (scooby) applied to pre-existing, publicly available single-cell multi-omics datasets (e.g., NeurIPS hematopoiesis dataset, OneK1K cohort, Heart organoid dataset)

Reporting for specific materials, systems and methods

We require information from authors about some types of materials, experimental systems and methods used in many studies. Here, indicate whether each material, system or method listed is relevant to your study. If you are not sure if a list item applies to your research, read the appropriate section before selecting a response.

Materials & experimental systems

n/a	Involved in the study
☒	☐ Antibodies
☒	☐ Eukaryotic cell lines
☒	☐ Palaeontology and archaeology
☒	☐ Animals and other organisms
☒	☐ Clinical data
☒	☐ Dual use research of concern
☒	☐ Plants

Methods

n/a	Involved in the study
☒	☐ ChIP-seq
☒	☐ Flow cytometry
☒	☐ MRI-based neuroimaging

Plants

Seed stocks	n/a
Novel plant genotypes	n/a
Authentication	n/a
